# Health-Related Quality of Life Before and After Sobriety in Combination With an Adjunctive Journaling App in Patients With Alcohol-Related Liver Disease: Prospective Single-Arm Study

**DOI:** 10.2196/80421

**Published:** 2026-03-05

**Authors:** Noriyo Yamashiki, Noriko Kudo, Kyoko Kawabata, Takako Fujimaki, Eiji Aramaki, Miki Murata, Shunichiro Ikeda, Hiroko Yoshii, Hisako Yamada, Makoto Naganuma

**Affiliations:** 1 Department of Gastroenterology and Hepatology Kansai Medical University Medical Center Moriguchi, Osaka Japan; 2 The Third Department of Internal Medicine Division of Gastroenterology and Hepatology Kansai Medical University Hirakata, Osaka Japan; 3 Graduate School of Science and Technology Nara Institute of Science and Technology Ikoma, Nara Japan; 4 Department of Psychiatry Kansai Medical University Moriguchi, Osaka Japan

**Keywords:** alcohol-related liver disease, health-related quality of life, patient-reported outcome, smartphone journaling app, SF-36

## Abstract

**Background:**

Alcohol-related liver disease (ALD) is a global health concern, and harmful alcohol use negatively affects clinical outcomes and health-related quality of life (HRQOL). Previous studies have demonstrated impaired HRQOL in chronic liver disease, including ALD; however, evidence remains limited regarding whether hepatologist-delivered abstinence support in outpatient practice can improve HRQOL. Digital interventions such as smartphone journaling apps may support behavior change, but their effectiveness in hepatology-led care settings has not been well established.

**Objective:**

This study aimed to prospectively evaluate changes in HRQOL over time among patients with ALD receiving routine hepatologist-led abstinence support supplemented by a smartphone-based journaling app.

**Methods:**

This was a prospective single-arm observational study. Eligibility criteria included a diagnosis of ALD, outpatient follow-up in hepatology, advice to abstain from alcohol, and written informed consent; patients were excluded if they reported abstinence for >1 year, had no intention to reduce or abstain from alcohol, or declined medical follow-up. In total, 21 outpatients with ALD (mean age 51, SD 3 years; men: n=12, 57.1%) were enrolled from a gastroenterology and hepatology outpatient clinic between August 2021 and July 2023 (convenience sampling). Participants attended scheduled visits at 4, 8, 12, and 24 weeks and received brief abstinence counseling by hepatologists while using a journaling app. HRQOL was assessed using the Japanese version of the 36-Item Short Form Health Survey version 2 at entry, week 8, and week 24. Changes in subscale and summary scores were analyzed using paired *t* tests (2-sided α=.05) with 95% CIs; when normality assumptions were not met, Wilcoxon signed-rank tests were applied.

**Results:**

From entry to week 8, significant improvements were observed in physical functioning (mean difference [MD] +4.70, 95% CI 1.62-7.78; *P*=.005), role physical (MD +7.87, 95% CI 1.05-14.70; *P*=.02), general health (MD +5.82, 95% CI 1.74-9.88; *P*=.007), and role emotional (MD +7.85, 95% CI 0.29-15.41; *P*=.04). The Physical Component Summary score also improved (MD +4.78, 95% CI 1.22-8.34; *P*=.01). These findings were consistent in sensitivity analysis using the Wilcoxon signed-rank test. No significant changes were observed in mental or social HRQOL domains. In subgroup analyses, patients who maintained abstinence during the first 8 weeks (7/21, 33.3%) showed continued improvement in physical HRQOL up to 24 weeks, whereas those who continued drinking (11/21, 52.4%) did not exhibit meaningful change.

**Conclusions:**

In this exploratory observational study, hepatologist-led abstinence support supplemented by a smartphone-based journaling app was associated with improvements in physical HRQOL. Unlike previous studies that have focused on addiction specialist–led interventions, this study evaluated abstinence support delivered by hepatologists in routine outpatient practice, providing a real-world assessment of a digitally supported approach. These findings suggest that hepatologist-led, digitally supported care may be a feasible strategy in clinical settings with limited access to specialized addiction services.

## Introduction

### Alcohol-Related Liver Disease and the Clinical Relevance for Abstinence Support

Alcohol-related liver disease (ALD) is a major and growing global health problem, with increasing prevalence and disease burden worldwide, including in Japan [1-4]. The World Health Organization adopted a global strategy to reduce the harmful use of alcohol in 2010 and, more recently, reinforced the urgency of this issue through the Global Alcohol Action Plan 2022-2030, underscoring that harmful alcohol use remains a major global priority [1]. Recent epidemiological data indicate that the prevalence and impact of ALD continue to rise worldwide, including in Japan [2-4].

ALD encompasses a wide spectrum of diseases, ranging from simple steatosis to cirrhosis and hepatocellular carcinoma, and ongoing alcohol consumption plays a central role in disease progression [5]. Observational studies have consistently reported associations between abstinence and favorable liver-related outcomes, positioning interventions targeting drinking behavior as a core component of ALD management [5-7].

Early identification of harmful drinking and timely intervention are therefore essential components of ALD management. Screening tools such as the Alcohol Use Disorders Identification Test are widely used in clinical practice [8,9], and brief interventions that encourage self-monitoring of alcohol consumption have been shown to reduce alcohol intake [10]. While such identification and early intervention are ideal, most patients with alcohol use disorder (AUD) have not received intervention or been referred to specialists [11]. A recent survey of hepatologists and gastroenterologists reported that although AUD screening was nearly universal, many clinicians reported low comfort with and low rates of prescribing AUD pharmacotherapy due to insufficient training and perceived knowledge gaps [12]. Collaboration with a multidisciplinary team may improve interventions for AUD [13]. Although evidence remains limited, recent randomized controlled trials have demonstrated that digital tools, including smartphone apps, can contribute to reductions in alcohol consumption [14].

### Evidence for Digital Interventions in Alcohol Use

Systematic reviews of mobile health interventions for alcohol use have reported that many mobile apps and digital strategies are associated with reductions in alcohol consumption or related behaviors, although evidence regarding optimal design and outcomes remains heterogeneous [15]. In addition, systematic reviews of telehealth and digitally delivered cognitive behavioral therapy for AUD suggest that digital tools can decrease drinking behavior and may offer sustained benefits when used longitudinally [16]. Despite this growing evidence, most digital alcohol interventions have been evaluated under controlled research conditions or in general populations rather than within routine hepatology outpatient practice [14,15]. Taken together, these studies suggest that digital tools are feasible and potentially effective for alcohol reduction, but evidence remains limited regarding their impact on patient-centered outcomes, such as health-related quality of life (HRQOL) in routine hepatology care.

Preliminary studies in patients with alcohol-associated liver disease have demonstrated the feasibility and potential utility of digital therapeutics that combine smartphone apps with behavior change techniques, reporting associations with reduced alcohol use and increased abstinence in open-label settings [17]. Furthermore, a multicenter randomized controlled trial protocol has been published to evaluate the real-world effectiveness and cost-effectiveness of such smartphone-based digital therapeutics in patients with ALD, highlighting a growing focus on real-world implementation [18]. A usability study also indicates that patients with ALD generally perceive mobile health tools as accessible and acceptable for supporting alcohol cessation, supporting their integration into routine care [19]. Self-monitoring of alcohol intake is a core component of many behavioral change interventions and has been associated with increased awareness of drinking patterns and motivation to reduce consumption [15]. In this context, we previously developed a smartphone-based journaling app designed to support abstinence through patient self-monitoring and hepatologist review. In our pilot study conducted in a hepatology outpatient clinic, this approach was associated with reduced alcohol consumption and improved liver function over an 8-week period [20]. However, the broader impact of such abstinence support on patient-centered outcomes remains unclear.

### HRQOL in ALD: Evidence Gaps in Routine Outpatient Care

Beyond biochemical and clinical outcomes, ALD substantially impairs patients’ quality of life (QOL). Harmful alcohol use is associated with multidimensional burdens affecting physical, psychological, and social functioning, and HRQOL is commonly evaluated using generic instruments such as the 36-Item Short Form Health Survey (SF-36) and EQ-5D [21-23]. Previous studies have shown that HRQOL is impaired in patients with chronic liver disease regardless of etiology [23] and that ongoing alcohol use in cirrhosis is associated with further deterioration in QOL [24].

More recent evidence from studies in patients with alcoholic hepatitis has further highlighted the prognostic and clinical relevance of HRQOL [25]. In these highly selected populations with advanced disease severity and poor short-term prognosis (90-day mortality was approximately 15%), lower baseline mental and physical component scores were associated with increased mortality, and patients who achieved sustained abstinence over 180 days demonstrated greater recovery in both mental and physical HRQOL compared with those who continued drinking [25].

However, these findings were derived from patients with advanced disease in inpatient or specialist settings. It remains unclear whether similar changes in HRQOL can be observed in routine hepatology outpatient practice, where patients with ALD are often managed by hepatologists rather than addiction specialists and where disease severity is generally less advanced.

### Study Rationale and Aim

In routine hepatology outpatient practice, hepatologists frequently provide abstinence guidance as part of longitudinal ALD management [5,26], yet the impact of such care on HRQOL has not been well characterized. Moreover, evidence remains limited regarding whether a simple, feasible digital tool—such as a journaling app integrated into routine visits—is associated with longitudinal changes in HRQOL in real-world hepatology settings. Understanding these effects is essential to optimize patient-centered care and to inform the integration of digital tools into routine hepatology practice. Therefore, we prospectively evaluated changes in HRQOL over time among patients with ALD receiving hepatologist-led abstinence support supplemented by a smartphone-based journaling app.

## Methods

### Inclusion and Exclusion Criteria

Patients were eligible if they (1) had a diagnosis of ALD, (2) were outpatients attending the gastroenterology and hepatology clinic, (3) had been advised to abstain from alcohol, and (4) provided written informed consent. Patients were excluded if they self-reported having already maintained abstinence for more than 1 year, reported no intention to reduce or abstain from alcohol intake, or declined medical follow-up.

### Participant Characteristics

A total of 21 adult outpatients with ALD were enrolled. The mean age was 51 years, and 57.1% (n=12) of the patients were men. All participants were receiving routine hepatology outpatient care and had been advised to abstain from alcohol.

### Sampling Procedures

Participants were recruited consecutively during routine outpatient clinic visits (convenience sampling) between August 2021 and July 2023. No randomization or stratification procedures were applied. During the recruitment period, 31 patients who were considered eligible were approached. Of these 31 patients, 21 (67.7%) provided written informed consent and were enrolled. In total, 10 (10/31, 32.3%) patients were not enrolled because 7 (70%) reported no intention to reduce or abstain from alcohol use and 3 (30%) declined medical follow-up. Patients attended scheduled outpatient visits at 4, 8, 12, and 24 weeks. All participants received brief abstinence counseling by hepatologists and used a journaling app; app use was required until the 8-week visit and voluntary thereafter.

### Sample Size, Power, and Precision

As HRQOL assessment was an exploratory outcome in this observational study, no a priori sample size or power calculation was performed. The sample size was therefore determined by the number of eligible patients who consented during the study period, and the analyses were intended to be descriptive and hypothesis generating rather than confirmatory. All analyses were conducted after completion of follow-up for all participants.

### Measures and Covariates

HRQOL was assessed as the primary outcome using the Japanese version of the SF-36 version 2 (SF-36v2) at entry, week 8, and week 24. Achieved abstinence was defined as self-reported alcohol intake of 0 throughout the observation period.

No additional covariates were prespecified, and all analyses were conducted descriptively.

### Data Collection

HRQOL data were collected using a self-administered questionnaire. Participants completed the Japanese version of the SF-36v2 independently at the outpatient clinic at entry, week 8, and week 24.

### Quality of Measurements

To enhance data quality, trained research staff reviewed the completed questionnaires at each visit to check for missing responses or duplicate entries. When incomplete or unclear responses were identified, participants were asked to confirm or complete the items at the same visit.

### Instrumentation

A smartphone-based journaling app with basic logging and clinician review functions was used [20]. App engagement metrics were not assessed because the primary outcome was HRQOL. HRQOL was assessed using the Japanese version of the SF-36v2 [27,28]. The SF-36 measures 8 health domains: physical functioning (PF), role physical (RP), bodily pain, general health (GH), vitality, social functioning, role emotional (RE), and mental health [29]. Three summary scores were derived from these subscales: the Physical Component Summary, Mental Component Summary (MCS), and Role/Social Component Summary (RCS). Scores were calculated using the official Japanese scoring system (web based) and expressed on a 0- to 100-point scale with norm-based scoring, standardized to a national mean of 50 (SD 10) based on the 2017 Japanese population norms [30].

### Masking

No masking or blinding procedures were implemented, as this was an observational study conducted during routine outpatient care.

### Psychometrics

The SF-36v2 is a widely used and validated generic measure of HRQOL [29,31]. The Japanese version has demonstrated good internal consistency, with Cronbach α coefficients generally ranging from 0.72 to 0.90 across subscales, and acceptable test-retest reliability (intraclass correlation coefficients approximately 0.65-0.85) [27]. Construct validity has been supported by expected correlations with clinical status and by known-group differences between patients with chronic diseases and the general population [28,30]. Factor analyses have also confirmed a 2-component structure corresponding to the Physical and Mental Component summaries, consistent with the original US version [28]. Norm-based scoring is standardized to a national mean of 50 (SD 10) based on representative Japanese population data [30]. Given the prior validation of this instrument, no additional psychometric validation was performed in this study.

### Conditions and Design

This study used a prospective, single-arm observational design. Participants were drawn from patients who had participated in a previously reported interventional study using a smartphone-based journaling app [20].

### Data Diagnostics

Descriptive statistics were used to summarize patient characteristics at entry, including counts, means, medians, SD, ranges, minimum, and maximum for continuous variables and percentages for categorical variables. Missingness patterns were evaluated using the Little missing completely at random (MCAR) test, applied only to variables with missing data. Normality of continuous variables was assessed using the Shapiro-Wilk test.

### Analytic Strategy

Changes in HRQOL scores were analyzed using paired *t* tests for each observed case, with mean differences and 95% CIs reported. For variables in which normality was rejected, the Wilcoxon signed-rank test was additionally performed. For subgroup analyses, due to the small number of participants, only the Wilcoxon signed-rank test was used. Between-group comparisons for characteristics at entry were conducted using Student *t* tests for normally distributed variables and the Wilcoxon rank sum test for nonparametric data. Categorical variables were compared using chi-square tests or Fisher exact tests, as appropriate. Summary scores were compared at entry, 8 weeks, and 24 weeks. A 2-sided *P* value of <.05 was considered statistically significant. All analyses were performed using STATA 18 (StataCorp LLC).

### Reporting Standards

This paper was written in accordance with the *American Psychological Association* (*APA*) style, including the *Journal Article Reporting Standards for Quantitative Research* (*JARS-Quan*) [32].

### Ethical Considerations

This study was registered with the University Hospital Medical Information Network Clinical Trials Registry (UMIN000045285). This study was approved by the Ethics Review Center of Kansai Medical University (2020252) and conducted in accordance with the Declaration of Helsinki. Written informed consent was obtained from all participants before enrollment. All data were deidentified before analysis, and no personally identifiable information was collected or stored. No compensation was provided to participants. The manuscript and supplementary materials contain no images or information that could identify individual participants.

## Results

### Patient Characteristics

Patient characteristics at entry are summarized in Table 1. The mean age was 51 (SD 3) years. Of the 21 patients, 12 (57.1%) were men, and 9 (42.9%) were women. The average BMI was 24.3 (SD 4.5) kg/m^2^. In total, 52.4% (n=11) of the patients were married, and 66.7% (n=14) were employed; 38.1% (n=8) had coexisting mental disorders, and 66.7% (n=14) were smokers. Regarding drinking history, the average age at onset of drinking was 19 (SD 1) years, with an average drinking duration of 28 (SD 10) years and a daily alcohol consumption of 112 (SD 72) g. The mean Alcohol Use Disorders Identification Test score was 20 (SD 9).

**Figure 1:**
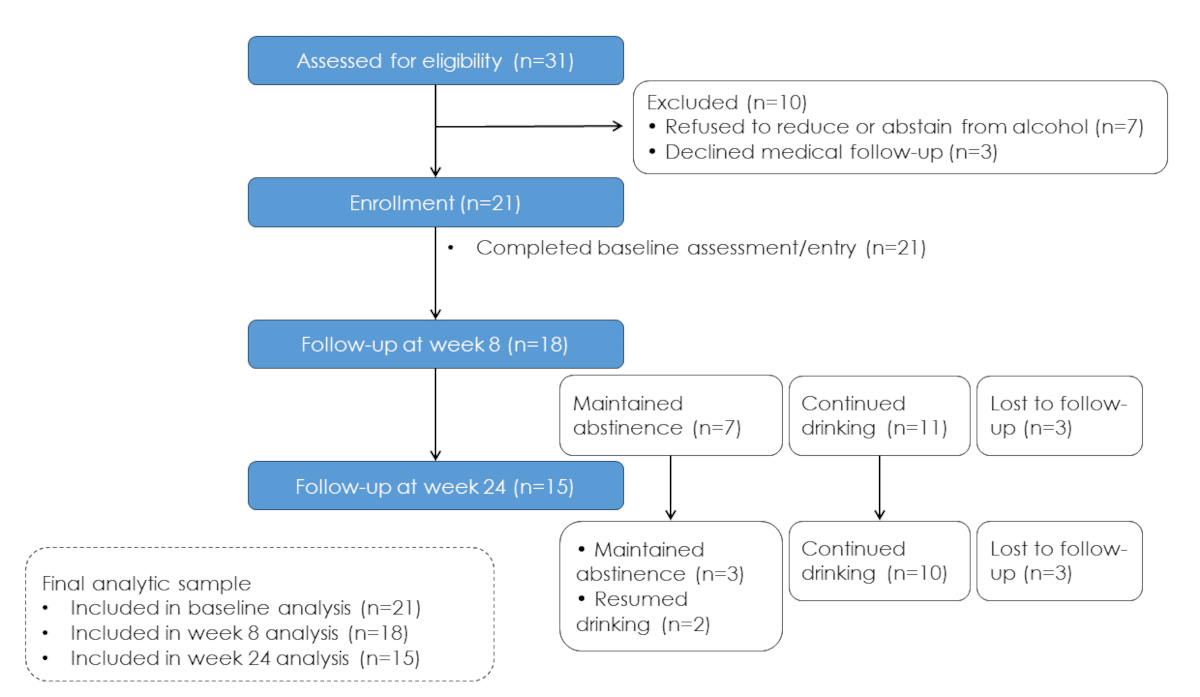
Participant flow diagram. In total, 31 patients were assessed for eligibility, of whom 21 were enrolled. Follow-up attendance included 18 patients at week 8 and 15 patients at week 24. At week 8, participants were classified into abstinence and nonabstinence groups based on self-reported alcohol intake.

Of the 21 patients enrolled, 18 (85.7%) remained in follow-up at 8 weeks, and 15 (71.4%) remained in follow-up at 24 weeks. Patient flow is shown in Figure 1. At 8 weeks, of the 18 patients, 7 (38.9%) who abstained were classified as the abstinence group, and 11 (61.1%) who continued drinking were classified as the nonabstinence group. At 24 weeks, 20% (3/15) of the patients remained abstinent. Here, the achieved abstinence was based on the self-reported alcohol intake of zero during the observation period.

Background factors are presented in Multimedia Appendix 1 by patient drinking status at 8 weeks. Patients who continued abstinence for 8 weeks had more liver cirrhosis than others (7/7, 100% vs 5/14, 35.7%; Fisher exact test *P*=.007). Similarly, more patients who continued abstinence for 8 weeks were hospitalized for liver-related complications (5/7, 71.4% vs 1/14, 7.1%; risk difference=−64.3%, 95% CI −100.4% to −28.2%; *P*=.002) and had a prolonged international normalized ratio (mean difference 0.55, 95% CI 0.26-0.84; *P*<.001) and lower hemoglobin (mean difference −2.58 g/dL, 95% CI −3.93 to −1.23; *P*<.001) and albumin (mean difference −1.1 g/dL, 95% CI −1.84 to −0.42; *P*=.004) than others.

**Table 1:** Patient characteristics at entry of 21 patients with alcohol-related liver disease enrolled in a prospective single-arm study.

Factor		Values
Age (y), mean (SD)		51 (3)
Male sex, n (%)		12 (57)
BMI^a^ (kg/m^2^), mean (SD)		24.3 (4.5)
Married or coresiding with others^a^, n (%)		11 (52.3)
Employed, n (%)		14 (66.6)
**Mental illness comorbidity, n (%)**		8 (38.1)
	Alcohol use disorder	2 (9.5)
	Alcohol use disorder and depression	2 (9.5)
	Depression	1 (4.7)
	Panic disorder	1 (4.7)
	Eating disorder	1 (4.7)
	Depression and eating disorder	1 (4.7)
**Smoking^a^** **, n (%)**		
	Current smoker	14 (66.6)
	Former smoker	3 (14.2)
	Nonsmoker	3 (14.2)
	Unknown	1 (4.7)
Age of starting drinking (y), median (IQR)		20 (16-20)
Drinking duration (y), mean (SD)		28 (10)
Daily alcohol consumption (ethanol equivalent; g), mean (SD)		112 (72)
AUDIT^b^ score at entry, mean (SD)		20 (9)

^a^Missing values were observed for BMI (n=1), cohabitation status (n=1), and smoking status (n=1).

^b^AUDIT: Alcohol Use Disorders Identification Test.

### Missing Data and MCAR Assessment

A small amount of missing data was present among the variables at entry: BMI (kg/m^2^; 1/21, 4.8%), cohabitation status (1/21, 4.8%), and smoking status (1/21, 4.8%). The Little MCAR test yielded *χ*^2^_6_=10.8 (*P*=.09), indicating that the pattern of missingness was consistent with MCAR.

### HRQOL Scores at Entry

**Figure 2:**
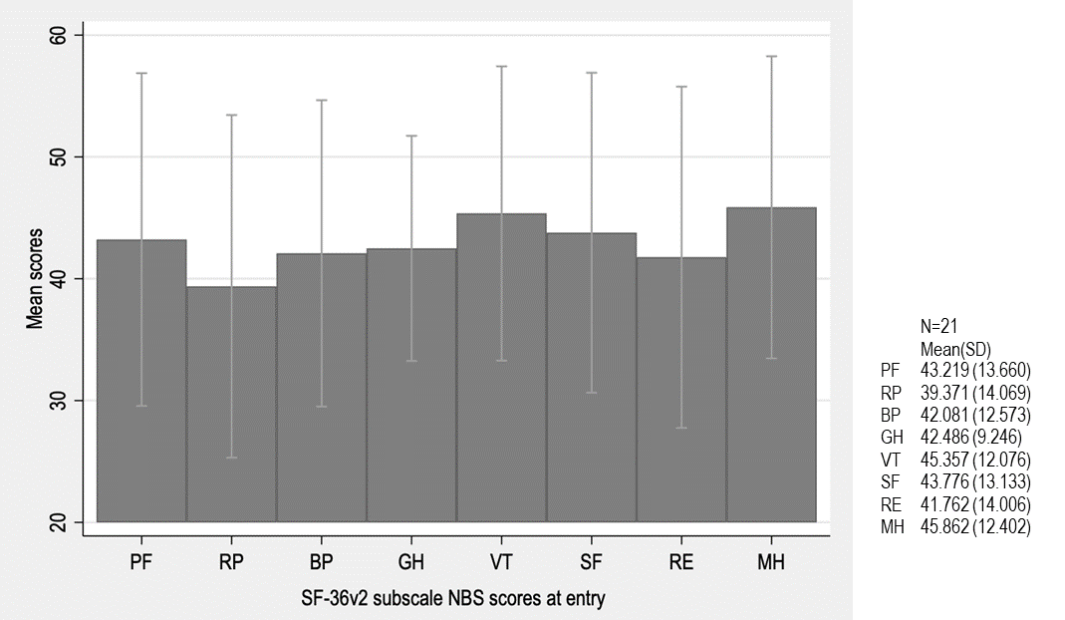
Mean scores of the 8 SF-36v2 subscales at entry, expressed using norm-based scoring (NBS). Scores are standardized to the Japanese population norm (mean 50, SD 10). Error bars indicate SD. Participants demonstrated lower scores across all 8 subscales compared with the Japanese population norms. BP: bodily pain; GH: general health; MH: mental health; PF: physical functioning; RE: role emotional; RP: role physical; SF: social functioning; VT: vitality.

The average scores of the 8 subscales assessed with SF-36v2 at entry are shown in Figure 2 (Multimedia Appendix 2). Patients enrolled in this study had lower HRQOL scores in all 8 subscales compared with the Japanese population norms (mean 50, SD 10 points).

### Changes in HRQOL Scores

**Figure 3:**
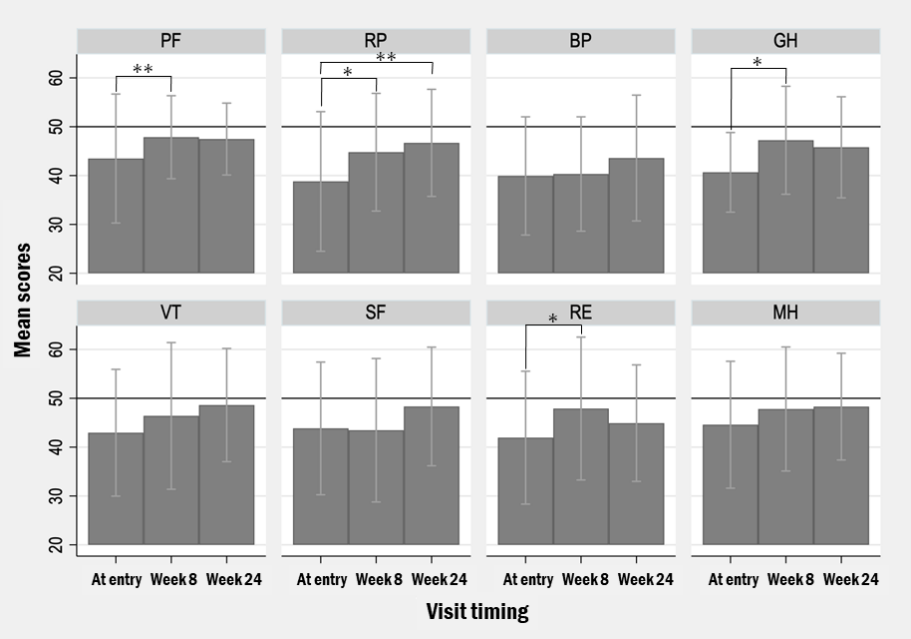
Changes in mean SF-36v2 subscale scores at entry, week 8, and week 24, expressed using norm-based scoring (NBS). Error bars indicate SD. Physical functioning (PF) increased by 4.70 points (95% CI 1.62-7.78; *P*=.005), role physical (RP) increased by 7.87 points (95% CI 1.05-14.70; *P*=.02), general health (GH) increased by 5.82 points (95% CI 1.74-9.88; *P*=.007), and role emotional (RE) increased by 7.85 points (95% CI 0.29-15.41; *P*=.04) after 8 weeks. BP: bodily pain; MH: mental health; SF: social functioning; VT: vitality. *P<.01; **P<.05.

**Figure 4:**
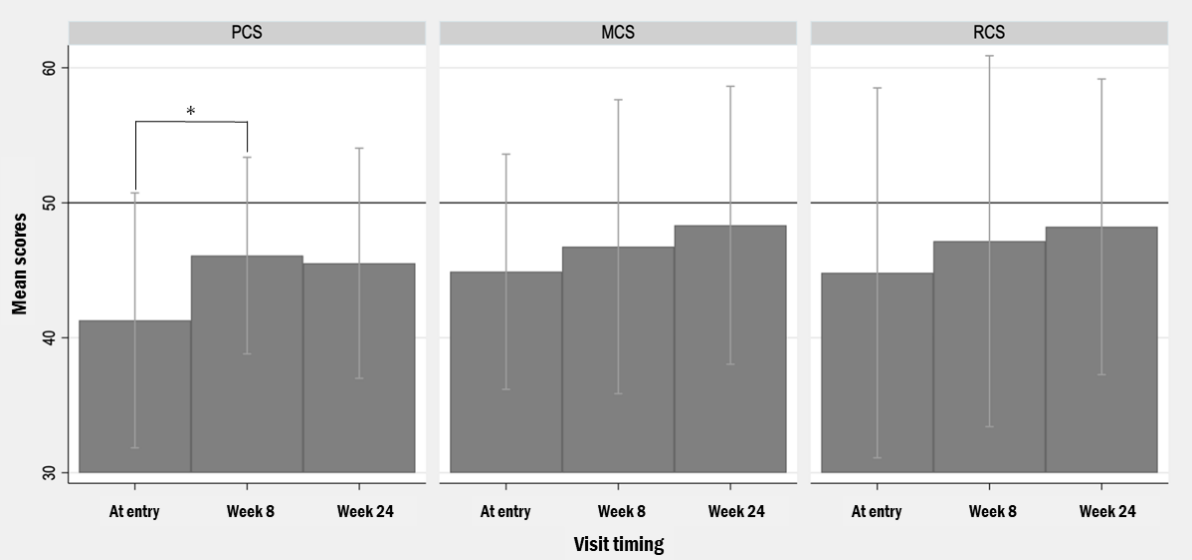
Changes in mean SF-36v2 component summary scores (physical, mental, and role or social) over time at entry, week 8, and week 24. Scores are presented using norm-based scoring (NBS). Error bars indicate SD. MCS: Mental Component Summary; PCS: Physical Component Summary; RCS: Role/Social Component Summary. *P<.05.

The results of pairwise comparison of each subscale and summary scores are shown in Figures 3 and 4. At the subscale level, PF increased by 4.70 points (95% CI 1.62-7.78; *P*=.005), RP increased by 7.87 points (95% CI 1.05-14.70; *P*=.02), GH increased by 5.82 points (95% CI 1.74-9.88; *P*=.007), and RE increased by 7.85 points (95% CI 0.29-15.41; *P*=.04) after 8 weeks (Figure 3). These results were consistent in a sensitivity analysis using the Wilcoxon signed-rank test (PF: *P*=.005; RP: *P*=.01; GH: *P*=.01; RE: *P*=.04). No significant changes were observed for bodily pain, vitality, social functioning, mental health, MCS, or RCS. The PCS score increased by 4.78 points (95% CI 1.22-8.34; *P*=.01), which was similarly supported by the Wilcoxon signed-rank test (*P*=.03; Figure 4). Changes in MCS and RCS were not significant on either test. From entry to week 24, RP showed a significant improvement, increasing by 7.90 points (95% CI 2.23-13.57; *P*=.009), which was consistent with the Wilcoxon signed-rank test (*P*=.02). In contrast, no other subscale or summary score showed significant changes from entry to week 24 or from week 8 to week 24 on 2-tailed paired *t* tests (all *P*>.14), and similar nonsignificant results were obtained using Wilcoxon signed-rank tests.

Further subgroup analysis revealed that patients in the abstinence group demonstrated significant improvements in PF (*P*=.02), GH (*P*=.02), and RE (*P*=.049) from entry to week 8 (Multimedia Appendix 3), whereas the nonabstinence group showed no appreciable improvement or deterioration (Multimedia Appendix 4). While overall pre-post changes in the entire cohort were analyzed using paired *t* tests, the subgroup comparisons were conducted using Wilcoxon signed-rank tests owing to the small sample size and deviation from normality.

## Discussion

### Principal Findings

In this study, we aimed to evaluate changes in HRQOL over time among patients with ALD receiving routine outpatient care by hepatologists supplemented by the use of a journaling app. Consistent with this objective, we observed that physical HRQOL improved over the follow-up period, particularly among patients who maintained abstinence, whereas mental and social HRQOL domains showed no meaningful change. These findings provide an overview of how HRQOL evolves in this cohort when hepatologists address abstinence in routine outpatient care and suggest which HRQOL domains tend to change over time.

Previous studies have consistently shown that the severity of liver cirrhosis is inversely associated with QOL, particularly in the physical domain [31], and that individuals who develop liver cirrhosis at a younger age tend to experience a greater decline in HRQOL [33]. In this study, HRQOL scores across all 8 SF-36v2 subscales were lower than the Japanese population norms at entry, which is likely explained by the fact that more than half (12/21, 57.1%) of the participants had cirrhosis. This finding is consistent with prior reports demonstrating substantially impaired HRQOL among patients with advanced liver disease or cirrhosis-related complications [23,24,34]. When HRQOL trajectories were examined over time, patients who maintained abstinence showed gradual improvement in physical aspects of HRQOL, whereas no meaningful improvement was observed among those who continued drinking. Notably, the abstinent group had lower HRQOL scores at entry and included a higher proportion of patients with cirrhosis or a history of recent hospitalization. This baseline imbalance represents a potential confounding factor, limiting the extent to which subsequent differences in HRQOL trajectories can be attributed solely to abstinence or the intervention itself. Although causal mechanisms cannot be determined in this observational study, one possible explanation is that patients with more advanced disease or recent symptoms may have had greater awareness of their impaired health status, which could have strengthened their motivation to abstain from alcohol. A previous study has reported that recently hospitalized patients with ALD showed increased motivation to reduce their alcohol consumption [35]. In contrast, patients who continued drinking or who dropped out may have perceived less immediate impact of their disease on daily functioning, potentially limiting both abstinence motivation and observable HRQOL improvement. In our previous report using the same cohort, abstinent patients demonstrated improvements in liver function during the early follow-up period [20]. Taken together, these findings may suggest that recovery of liver function associated with abstinence may contribute to subsequent improvements in physical aspects of HRQOL, even among patients with impaired baseline liver function. Although the foregoing interpretation remains speculative, the observed pattern is not inconsistent with previous reports linking disease severity, abstinence, and physical HRQOL in chronic liver disease [22-24,31].

Whether changes in HRQOL during abstinence interventions are clinically meaningful has not been well characterized in previous studies. A recent observational study focusing on patients with alcoholic hepatitis reported comparable findings in a population with more severe liver dysfunction [25]. In that study, patients with alcoholic hepatitis exhibited substantially lower physical HRQOL scores at baseline than heavy drinkers without hepatitis, reflecting advanced disease severity. Importantly, lower baseline HRQOL was associated with poor prognosis independently of the Model for End-Stage Liver Disease score, and improvements in HRQOL were linked to both abstinence and improvement in liver function [25]. This study shares several key features with these findings, including the coexistence of impaired baseline liver reserve and reduced HRQOL, as well as the association between sustained abstinence and subsequent improvement in physical HRQOL. However, the population studied by Madathanapalli et al [25] consisted of patients with a very high short-term mortality risk. This represents a substantially more severe disease spectrum than that typically encountered in routine outpatient hepatology practice. Although limited by sample size, our study extends these observations by demonstrating similar HRQOL trajectories in a real-world outpatient cohort of patients with ALD, many of whom had cirrhosis but were clinically stable enough to be managed in an ambulatory setting. This suggests that the relationship between abstinence, liver function recovery, and improvement in physical HRQOL may also be relevant in less severely ill patients routinely followed by hepatologists.

In this outpatient setting, abstinence support was provided by hepatologists through regular clinical follow-up combined with review of patients’ entries in the journaling app. The important role of hepatologists in the identification and management of AUD among patients with ALD has been emphasized in current clinical practice guidelines [5,26]. Although all participating hepatologists had completed e-learning modules on the diagnosis and management of alcohol dependence, they were not psychiatric or addiction specialists. Accordingly, the association observed between hepatologist-led follow-up and changes in HRQOL should be interpreted with caution. Nevertheless, regular outpatient contact and structured review of self-reported alcohol-related behaviors may have facilitated sustained patient engagement in abstinence efforts, which could have contributed to the observed stabilization and improvement of physical HRQOL. This interpretation is consistent with previous studies demonstrating that brief interventions delivered in general medical settings can positively influence alcohol use behaviors, even when provided by nonspecialists [10,14]. In contrast, improvements in mental and social domains of HRQOL were limited in this study. Previous studies have reported that psychological distress, stigmatization, and social isolation often persist in patients with alcohol-related conditions despite improvements in physical health and may not be sufficiently addressed by brief medical interventions alone [23]. This finding suggests that while hepatologist-led abstinence support may be sufficient to influence physical aspects of HRQOL, broader improvements in psychological and social well-being may require more comprehensive psychosocial or psychiatric interventions beyond the scope of routine hepatology outpatient care.

### Limitations

This study has several limitations. First, the small sample size and the absence of a control group limit the generalizability of the findings and preclude causal inference; therefore, the findings should be interpreted as exploratory. Baseline disease severity differed between the abstinent and nonabstinent groups, with the abstinent group showing more advanced liver disease at entry. This imbalance represents a potential confounding factor and limits causal interpretation, as observed improvements in HRQOL may partly reflect regression to the mean or greater potential for recovery rather than the effect of abstinence support alone. In addition, participant attrition was substantial, with only 85.7% (18/21) of the enrolled participants remaining in follow-up at 8 weeks and 71.4% (15/21) remaining in follow-up at 24 weeks, which may introduce attrition bias and further limit the robustness of longitudinal comparisons. Multiple statistical tests were performed without formal correction for multiple comparisons, which increases the risk of type 1 error. Therefore, statistically significant findings should be interpreted with caution. Second, engagement metrics for the journaling app (such as frequency or duration of use) were not collected or analyzed, as the primary outcome of this study was health-related QOL; thus, the relationship between intensity of app use and outcomes could not be evaluated. Third, because only the generic SF-36v2 was used, disease-specific psychosocial domains such as sleep disturbance, stigma, and social isolation may not have been fully captured.

The proposed app has the potential to evolve into a form of personal health record that integrates artificial intelligence functions to enhance patient motivation. However, legal guidelines defining safe feedback to patients remain unclear. Therefore, the app is currently limited to simple functions. Furthermore, the digital divide remains a serious concern, as older individuals or those with visual impairment may not be able to use the app, which may limit the broader applicability of this approach.

### Conclusions

In conclusion, hepatologist-led review of entries from a smartphone-based journaling app during routine outpatient visits was associated with improvements in the physical component of HRQOL among patients with ALD who achieved and maintained abstinence.

Unlike previous studies that have primarily examined abstinence interventions delivered by addiction specialists, this study evaluated an abstinence support approach integrated into routine hepatology practice and supplemented by a simple digital tool, an area in which evidence has been limited. By focusing on HRQOL outcomes, this study provides preliminary evidence that hepatologist-led, digitally supported abstinence care may contribute to improving physical well-being in patients with ALD.

From a broader perspective, these findings suggest that such an approach may represent a feasible real-world strategy in clinical settings where access to specialized addiction services is limited. Although further controlled and comparative studies are required, hepatologist-led, digitally supported abstinence support may help address existing care gaps in routine practice, while improvements in mental and social HRQOL likely require additional psychosocial or psychiatric interventions.

## Appendix

Multimedia Appendix 1 Patient background characteristics stratified by drinking status at 8 weeks. Patients who maintained abstinence at week 8 had a higher prevalence of liver cirrhosis compared with those who continued drinking.

Multimedia Appendix 2 Mean 36-Item Short Form Health Survey version 2 (SF-36v2) subscale scores at entry. At study entry, participants demonstrated lower health-related quality of life scores across all 8 subscales compared with Japanese population norms.

Multimedia Appendix 3 Changes in mean 36-Item Short Form Health Survey version 2 (SF-36v2) subscale scores at entry, week 8, and week 24 in the abstinence group, expressed using norm-based scoring (NBS). Error bars indicate SD. Subgroup comparisons were performed using Wilcoxon signed-rank tests because of the small sample size and nonnormal distribution.

Multimedia Appendix 4 Changes in mean 36-Item Short Form Health Survey version 2 (SF-36v2) subscale scores at entry, week 8, and week 24 in the nonabstinence group, expressed using norm-based scoring (NBS). Error bars indicate SD. The nonabstinence group showed no appreciable improvement or deterioration. The subgroup comparisons were conducted using Wilcoxon signed-rank tests owing to the small sample size and deviation from normality.

## Abbreviations

ALD: alcohol-related liver disease
APA: American Psychological Association
AUD: alcohol use disorder
GH: general health
HRQOL: health-related quality of life
JARS-Quan: Journal Article Reporting Standards for Quantitative Research
MCAR: missing completely at random
MCS: Mental Component Summary
MD: mean difference
PF: physical functioning
QOL: quality of life
RCS: Role/Social Component Summary
RE: role emotional
RP: role physical
SF-36: 36-Item Short Form Health Survey
SF-36v2: 36-Item Short Form Health Survey version 2

## Data Availability

The data supporting the findings of this study are not publicly available due to ethical and privacy restrictions but are available from the corresponding author upon reasonable request.
