# Supplementary material for: Health-Related Quality of Life Before and After Sobriety in Combination With an Adjunctive Journaling App in Patients With Alcohol-Related Liver Disease: Prospective Single-Arm Study
Source: JMIR Form Res. 2026 Mar 5;10:e80421. doi: 10.2196/80421 (PMC13003206; doi:10.2196/80421)
Supplement: Multimedia Appendix 1 [file formative_v10i1e80421_app1.docx]

Table S1. Patient background characteristics stratified by drinking status at 8 weeks. Patients who maintained abstinence at week 8 had a higher prevalence of liver cirrhosis compared with those who continued drinking.

| Factor | Continue abstinence (*n*=7) | Drinking or dropout (*n*=14) | *P* value |
| --- | --- | --- | --- |
| Age (years), mean (SD) | 51 (8) | 51 (10) | .92 |
| Male gender, *n* (%) | 4 (57) | 8 (57) | 1.00 |
| Cirrhosis, *n* (%) | 7 (100) | 5 (35) | .007 |
| Hospitalization for liver-related complications  before entry, n (%) | 5 (71) | 1 (7) | .006 |
| Married or Co-residing with other,  *n* (%) | 5 (57) | 7 (53) | .88 |
| Employed, *n* (%) | 5 (71) | 9 (64) | .74 |
| Diagnosed with a mental illness,  *n* (%) | 1 (14) | 7 (50) | .11 |
| Current smoking, *n* (%) | 6 (85) | 8 (57) | .19 |
| AUDIT score at entry, mean (SD) | 17 (12) | 22 (7) | .20 |
| INR, mean (SD) | 1.6 (0.4) | 1.1 (0.2) | <.001 |
| Hemoglobin (g/dL), mean (SD) | 10.5 (0.9) | 13.1 (1.5) | <.001 |
| AST (U/L), mean (SD) | 57 (22) | 73 (45) | .40 |
| ALT (U/L), mean (SD) | 52 (58) | 40 (27) | .50 |
| GGT (U/L), mean (SD) | 130 (131) | 424 (422) | .09 |
| TBil (mg/dL), mean (SD) | 4.0 (2.6) | 2.2 (4.0) | .28 |
| Albumin (g/dL), mean (SD) | 2.5 (0.8) | 3.6 (0.7) | .004 |
