# Supplementary material for: Health-Related Quality of Life Before and After Sobriety in Combination With an Adjunctive Journaling App in Patients With Alcohol-Related Liver Disease: Prospective Single-Arm Study
Source: JMIR Form Res. 2026 Mar 5;10:e80421. doi: 10.2196/80421 (PMC13003206; doi:10.2196/80421)
Supplement: Multimedia Appendix 2 [file formative_v10i1e80421_app2.docx]

Table S2. Mean SF-36v2 subscale scores at entry. At study entry, participants demonstrated lower health-related quality of life scores across all eight subscales compared with Japanese population norms.

| Scale | total (*n* =21) | male (*n* =12) | female (*n* =9) |
| --- | --- | --- | --- |
|  | mean (SD) | mean (SD) | mean (SD) |
| Physical functioning (PF) | 43.219 (13.660) | 45.075 (13.079) | 40.744 (14.805) |
| Role physical (RP) | 39.371 (14.069) | 43.233 (10.969) | 34.222 (16.648) |
| Bodily pain (BP) | 42.081 (12.573) | 42.508 (12.820) | 41.511 (12.983) |
| General health (GH) | 42.486 (9.246) | 43.092 (9.337) | 41.678 (9.620) |
| Vitality (VT) | 45.357 (12.076) | 44.775 (11.106) | 46.133 (13.921) |
| Social functioning (SF) | 43.776 (13.133) | 45.517 (13.093) | 41.456 (13.597) |
| Role emotional (RE) | 41.762 (14.006) | 41.925 (11.303) | 41.544 (17.739) |
| Mental health (MH) | 45.862 (12.402) | 46.100 (12.156) | 45.544 (13.458) |
