# Supplementary material for: Health-Related Quality of Life Before and After Sobriety in Combination With an Adjunctive Journaling App in Patients With Alcohol-Related Liver Disease: Prospective Single-Arm Study
Source: JMIR Form Res. 2026 Mar 5;10:e80421. doi: 10.2196/80421 (PMC13003206; doi:10.2196/80421)
Supplement: Multimedia Appendix 3 [file formative_v10i1e80421_app3.docx]

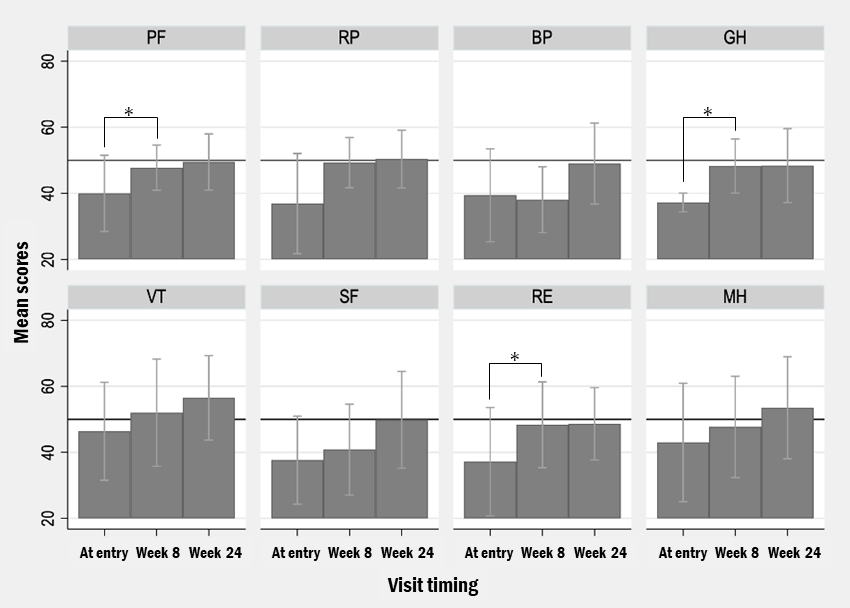


Multimedia Appendix 3. Changes in mean SF-36v2 subscale scores at entry, week 8, and week 24 in the abstinence group, expressed using norm-based scoring (NBS). Error bars indicate standard deviation. Subgroup comparisons were performed using Wilcoxon signed-rank tests because of the small sample size and non-normal distribution. **P*<.05.
